# Topological and functional characterization of human translation efficiency covariation network

**DOI:** 10.1093/bioinformatics/btaf583

**Published:** 2025-10-22

**Authors:** Kangsheng Qi, Can Cenik

**Affiliations:** Department of Molecular Biosciences, University of Texas at Austin, Austin, TX 78712, United States; Department of Molecular Biosciences, University of Texas at Austin, Austin, TX 78712, United States

## Abstract

**Motivation:**

Gene co-expression networks based on RNA abundance have identified genes with shared biological functions, common regulatory elements, and physical interactions among their protein products. Although thousands of ribosome profiling datasets are publicly available, they have not been leveraged to construct networks to characterize translation efficiency covariation (TEC) to quantify how translation of different transcripts co-varies across conditions.

**Results:**

We construct and analyze a human TEC network, revealing topological and functional properties distinct from RNA co-expression networks. The TEC network displays modular structure, small-world characteristics, and rich-club organization but differs substantially in node connectivity and neighborhood composition. Comparative analyses show that genes such as PKM, which are central in the TEC network due to their role in translational regulation, are peripheral in RNA co-expression networks. Tissue-specific TEC networks further uncover context-dependent translation patterns. These results suggest that TEC networks provide a complementary framework for understanding gene regulation.

**Availability and implementation:**

The code for this study is archived on https://zenodo.org/records/17275939 and publicly available at https://github.com/CenikLab/TEC-Network-Analyses. The associated data can be accessed at https://zenodo.org/records/17275970.

## 1 Introduction

RNA co-expression measures the similarity in transcript abundance between genes across conditions, thus revealing groups of genes with shared expression patterns. This information can be naturally formulated as a mathematical graph, where nodes correspond to genes and edges denote co-expression strengths (Langfelder and Horvath 2008, Mao *et al.* 2009, Kuang *et al.* 2022). Such graphs are commonly referred to as gene co-expression networks (GCNs).

By applying mathematical graph metrics and analysis techniques, such as degree distribution modeling, centrality measures, and clustering algorithms, GCNs have revealed that co-expressed genes tend to share functional annotations (Stuart *et al.* 2003, Bergmann *et al.* 2004, Mao *et al.* 2009, Kuang *et al.* 2022), exhibit common regulatory mechanisms (Stuart *et al.* 2003, Allocco *et al.* 2004, M Ribeiro *et al.* 2022), and be enriched for physically interacting pairs of proteins (Jansen *et al.* 2002, Szklarczyk *et al.* 2023). These findings established the utility of GCNs as data-driven representations of the cell’s underlying functional and regulatory architecture.

Beyond providing insights into the organization of cellular systems, GCNs can facilitate inferring functions of uncharacterized genes. Traditionally, this has been done using guilt by association paradigms, where a gene’s functions are inferred from those of its neighboring genes within the network (Stuart *et al.* 2003). More recently, this concept has been extended through graph neural networks and other machine learning models, which predict gene functions by aggregating vectorized gene representations guided by the structure of the GCN (You *et al.* 2021).

While many studies explored RNA co-expression data, the coordination of post-transcriptional regulation across transcripts has been much less characterized. This gap motivates the exploration of co-expression relationships beyond the transcriptome, particularly at the level of translation. We recently introduced the concept of ‘translation efficiency covariation’ (TEC) (Liu *et al.* 2025), which refers to the coordinated variation in the rate at which different transcripts are translated (translation efficiency) across cell types, as revealed by analyzing 1076 human and 845 mouse samples of paired ribosome profiling and RNA sequencing data. This coordinated behavior at the translation level is evolutionarily conserved between human and mouse. Biologically, TEC enables the prediction of novel gene functions, reveals potential physical interactions among proteins, and serves as an organizing principle of mammalian gene expression.

To the best of our knowledge, there are no previous studies that use graph-based approaches on networks that have been constructed to model coordinated patterns specifically at the level of translation. In this study, we present a graph-based framework to analyze TEC. We construct and characterize a human TEC network using graph-theoretic metrics and comparisons to canonical network models. Our results reveal that the TEC network is topologically distinct from its RNA co-expression counterpart and provides complementary and unique insights not captured by transcription level analyses alone. Lastly, we show that tissue-specific TEC networks reveal context-dependent co-translational features that are not observed in global analyses.

## 2 Methods

### 2.1 TEC and RNA co-expression data and network construction

We briefly summarize the methods used to compute TEC and RNA co-expression data, which follow the procedures described in Liu *et al.* (2025). We refer interested readers to the original publication for comprehensive details. All data were obtained from Ribobase (Liu *et al.* 2025), a curated and quality-controlled repository of RNA sequencing (RNA-seq) and ribosome profiling datasets from the Gene Expression Omnibus database processed using RiboFlow (Ozadam *et al.* 2020). For the global network, samples with matched RNA-seq and ribosome profiling data were used to estimate translation efficiency (TE) via compositional linear regression. TEC values were then derived as proportionality scores from cell-line level TE estimates using the propr package (lr2rho) (Quinn *et al.* 2017). RNA co-expression values were generated using a similar pipeline.

For tissue-specific networks, an additional filtering step based on tissue of origin was applied prior to estimating TE. For the liver-specific network, the cell lines included HCC tumor, HCC adjacent normal, HepG2, Huh-7.5, and Huh7. For the lung-specific network, cell lines included 12 T, A549, Calu-3, H1933, PC9, and WI38. For the brain-specific network, cell lines included U-251, U-343, early neurons, human brain tumor, neuronal precursor cells, neurons, and normal brain tissue.

Tissue-specific TEC calculation was performed using sample-level TE estimates. Finally, to construct the networks, genes were filtered to ensure a one-to-one mapping with UniProt accession numbers (UniProt Consortium 2025). Each gene was represented as a node, and the pairwise RNA or TEC scores were defined as edge weights between nodes.

### 2.2 Generation of mathematical network models

The Barabasi–Albert (BA) model (Barabasi and Albert 1999) was constructed using a preferential attachment process. To generate a BA model with *n* nodes, the parameter *m* was set to the edge-to-vertex ratio of the TEC network. The BA model was initialized with m+1 nodes organized in a star topology. New nodes were then added iteratively, each forming *m* edges to existing nodes with a probability proportional to their degree, thus favoring attachment to more connected nodes. This process was repeated until the network consisted of *n* nodes.

The Watts–Strogatz (WS) model (Watts and Strogatz 1998) with *n* nodes was generated by setting the parameter *k* to the average node degree of the TEC network. The model was initialized as a ring lattice where each node was connected to its *k* nearest neighbors. Subsequently, each edge was rewired with a probability of 0.01 (a tunable parameter). Given a degree sequence $\left\{d_{1},d_{2},\ldots ,d_{n}\right\}$, the expected degree random graph model (Chung and Lu 2002) was generated by defining the probability of an edge forming between nodes *u* and *v* as $d_{u}d_{v}/\sum_{i=1}^{n}d_{i}$. This enables the network to match the given degree distribution in expectation.

### 2.3 Scale-free analysis

We adopted the statistical framework proposed by Broido and Clauset (2019) and Clauset *et al.* (2009). Interested readers can refer to the original publications for further details.

To identify the optimal starting degree $k_{\min^{*}}$ for fitting a power law model to the TEC network, we computed the α exponent for a range of candidate $k_{\min}$ values using maximum likelihood estimation and evaluated their fit through the Kolmogorov–Smirnov (KS) *D* statistic. To assess the plausibility of the power law model, we generated 1000 synthetic distributions. For each synthetic dataset, we sampled *n* values independently from the fitted power law with probability $\frac{n_{\text{tail}}}{n}$ or uniformly from the network’s degree distribution below $k_{\min^{*}}$ with probability $1-\frac{n_{\text{tail}}}{n}$, where $n_{\text{tail}}$ is the number of nodes with degrees greater than or equal to $k_{\min^{*}}$. Each synthetic distribution was then fitted to its own power law, and we calculated the $p_{\text{fit}}$ value as the fraction of synthetic distributions with a worse KS *D* statistic than that of the observed data. We reject the power law as a plausible model if the $p_{\text{fit}}$ value is <0.1, indicating that over 90% of the synthetic distributions demonstrated a better fit.

Lastly, we compared the power law model to three alternative distributions: exponential, positive log-normal, and truncated power law ($p(k)∼k^{-\alpha }e^{-\gamma k}$), using the log-likelihood ratio test.

### 2.4 Graph metrics calculation

Given a node *u*, its clustering coefficient in an unweighted, undirected graph is defined as


(1) $$\text{clustering}(u)=\frac{2\cdot Tr(u)}{\deg(u)(\deg(u)-1)},$$

where Tr(u) is the number of triangles that pass through *u*, and deg(u) is the node degree of *u*. For a weighted, undirected graph, the clustering coefficient is modified to account for edge weights. In this case, it is defined as


(2) $$\text{clustering}(u)=\frac{1}{\deg(u)(\deg(u)-1)}\sum_{v,w\in N(u)}{\left(w_{u,v}w_{u,w}w_{w,v}\right)}^{\frac{1}{3}},$$

where the summation is over all pairs of neighbors *v* and *w* of *u* that form a triangle with *u*, and $w_{i,j}$ represents the weight of edge (i,j). This formulation is a natural interpretation as the unweighted clustering coefficient of the node scaled by the average intensity of its triangles (Onnela *et al.* 2005, Hagberg *et al.* 2008).

The rich-club coefficient (McAuley *et al.* 2007) for degree *k* is defined as


(3) $$\phi (k)=\frac{2E_{>k}}{N_{>k}(N_{>k}-1)},$$

where $E_{>k}$ is the number of edges between nodes with degrees >*k* and $N_{>k}$ is the number of such nodes.

To evaluate the similarity of the neighbors of each overlapping node *u*, we computed the Jaccard index:


(4) $$J(u_{\text{TEC}},u_{\text{RNA}})=\frac{\left|N(u_{\text{TEC}})\cap N(u_{\text{RNA}})\right|}{\left|N(u_{\text{TEC}})\cup N(u_{\text{RNA}})\right|},$$

where N(u) denotes the set of neighbors of node *u* in the respective network. Lastly, the density of a network is calculated


(5) $$d=\frac{2m}{n(n-1)},$$

where *n* is the number of nodes and *m* is the number of edges in the network.

### 2.5 Centrality calculation

We quantified node importance using three centrality measures: node strength (weighted degree centrality), closeness centrality (Freeman 2002), and betweenness centrality (Brandes 2001). All computations were done using NetworkX 3.5 (Hagberg *et al.* 2008). The centrality measures are defined as follows:


(6) $$\text{node}\;\text{strength}(u)=\sum_{v\in N(u)}w_{u,v},$$

where $w_{u,v}$ is the weight of edge (u,v).


(7) $$\text{closeness}(u)=\frac{1}{\sum_{u\neq v\in V}d(u,v)}(n-1),$$

where d(u,v) is the shortest path distance by edge weights between nodes *u* and *v*, and *n* is the total number of nodes in the network.


(8) $$\text{betweenness}(u)=\sum_{v,w\in V}\frac{\Gamma (v,w|u)}{\Gamma (v,w)},$$

where Γ(v,w) is the number of shortest paths between *u* and *w* and Γ(v,w|u) is the number of those paths which include *u*.

## 3 Results

### 3.1 Characterization of TEC network

TE, calculated ribosome-protected footprints normalized to mRNA abundance, approximates how effectively transcripts are converted into proteins and correlates significantly with protein abundance. The TEC metric captures coordinated TE patterns across cell types by computing the proportionality rho score between transcripts’ TE values across cell lines (Section 2). TEC values range from −1 to 1, reflecting either positive or negative covariation between a pair of transcripts. Both directions could indicate shared regulation or functional relation between transcripts, and consequently, we used the absolute value of TEC in this study.

**Figure 1.**
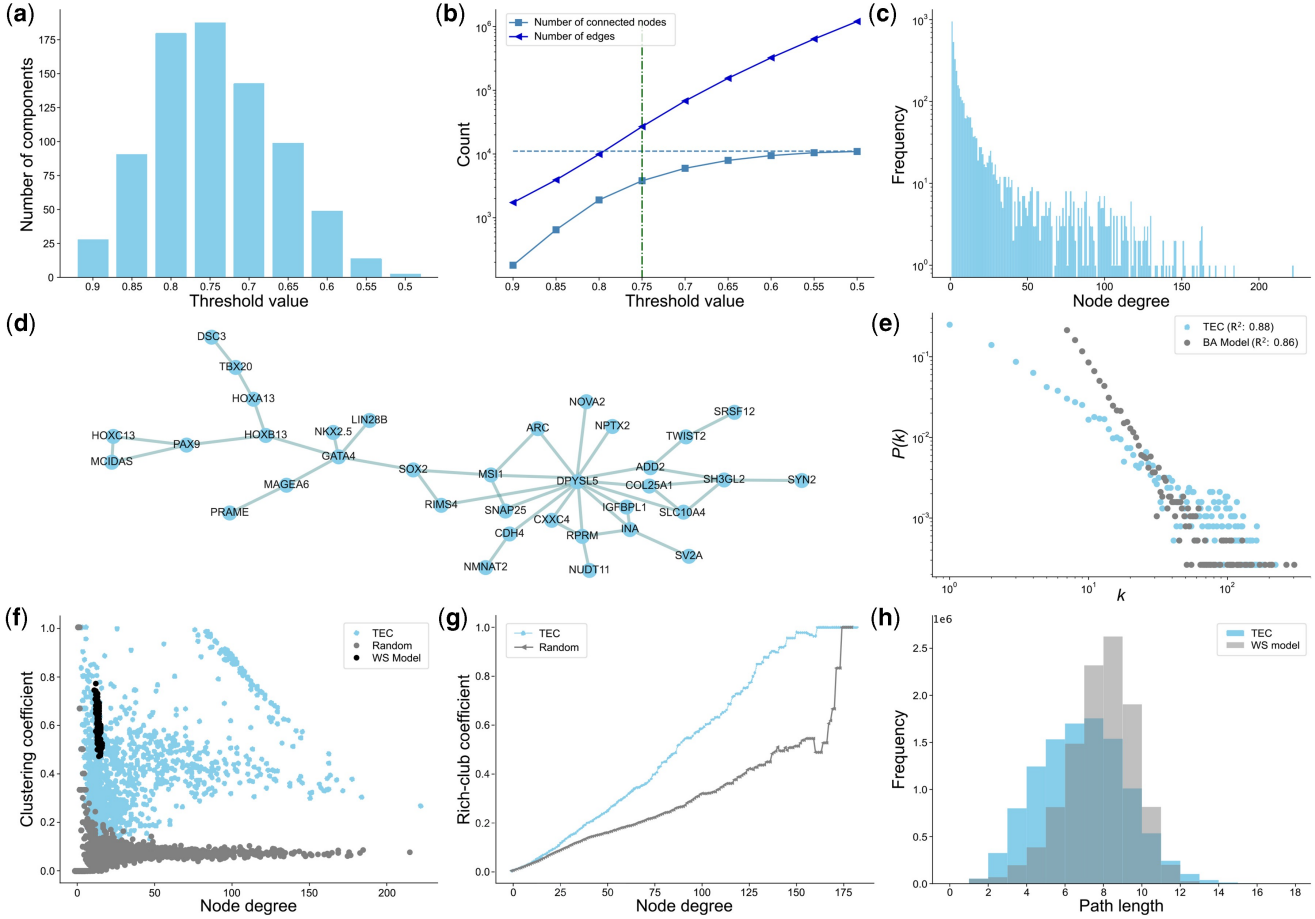
Characterization of TEC network. (a) Number of multi-node connected components as a function of edge weight threshold. (b) Number of connected nodes (light blue squares) and edges (dark blue triangles) as a function of the TEC threshold. The *y*-axis is log-scaled. (c) Degree distribution of the TEC network after removing isolated nodes using a cutoff of 0.75. The *y*-axis is log-scaled to account for variability in node connectivity. (d) Visualization of the second largest connected component using the Kamada–Kawai force-directed layout (Kamada and Kawai 1989). Each node is labeled with its corresponding gene symbol. (e) Assessment of scale-free properties by fitting a linear model to the log–log plot of degree *k* versus the probability of observing degree *k*, P(k), and comparing it to a BA model. Each dot represents a unique degree and its probability. (f) Comparison of node-level clustering coefficients in the TEC network against a random network with similar degree distribution (gray) and a WS model (black). Each dot represents a node, plotted by its degree and clustering coefficient. (g) Comparison of degree-level rich-club clustering coefficients in the TEC network against a random network with similar degree distribution (gray). (h) Distribution of shortest path lengths between all node pairs in the TEC network compared to the WS model (gray).

To determine an appropriate threshold for retaining biologically meaningful edges, we examined the number of multi-node connected components as a function of edge weight threshold (Fig. 1a). Below a threshold of 0.75, the number of multi-node components, potentially representing groups of functionally related genes, began to decrease. This suggests that existing components were merging more rapidly than new clusters were forming from isolated nodes.

We next analyzed the number of connected nodes and edges across varying thresholds (Fig. 1b). At higher thresholds, the network remained sparse, with over half of the nodes being isolated. As the threshold decreased, the number of edges increased exponentially, while the number of connected nodes rose rapidly before plateauing around a threshold of 0.60, where most nodes became reachable within the network.

The number of multi-node connected components peaked at a threshold of 0.75, suggesting a balance between sparsity and functional modularity. Based on this, we selected 0.75 as the cutoff for subsequent analyses, interpreting values above this threshold as indicative of strong covariation. To enable comparison against random network models, we binarized the network and removed isolated nodes, resulting in an unweighted graph with 3804 nodes and 26 878 edges.

The degree distribution of the resulting network revealed substantial heterogeneity in node connectivity with most nodes having relatively few edges and a small subset serving as highly connected hubs (Fig. 1c and d). This skewed distribution could indicate robustness to random perturbations (Albert *et al.* 2000) and hints at the presence of network organization with hierarchical features (Ravasz and Barabási 2003).

The long-tailed nature of the distribution suggests a resemblance to scale-free networks, which are characterized by a degree probability distribution that follows a power law with an α exponent between 2 and 3 (Barabasi and Albert 1999, Broido and Clauset 2019). To assess this quantitatively, we computed the degree probability distribution P(k) and performed a linear regression on its log–log transformation. As a reference, we generated a BA model, a canonical scale-free network, calibrated to match the number of nodes and approximate the edge density of the TEC network (Section 2). In a true scale-free network, this log–log plot of P(k) versus *k* should show strong linearity. The TEC network yielded a slightly higher coefficient of determination ($R^{2}=0.88$) than the BA model ($R^{2}=0.86$), despite the latter being explicitly designed to produce scale-free behavior (Fig. 1e). This likely stems from limitations of the log–log regression approach, which has been criticized to be sensitive to noise and lacks statistical rigor (Clauset *et al.* 2009, Stumpf and Porter 2012). A more rigorous evaluation (Section 2) rejected the power law as a plausible model for the TEC network. Moreover, its degree distribution was better described by a log-normal model. In contrast, the BA model was well-fitted by the power law, and alternative distributions showed poorer fits (Table 1, available as supplementary data at *Bioinformatics* online).

Having assessed global degree-based properties, we next examined the structural organization of the TEC network. High clustering tendency, where neighbors of a node tend to be interconnected, is commonly observed in biological networks and is theorized to be evolutionarily advantageous (Watts and Strogatz 1998, Wagner and Fell 2001). To assess this property, we computed the local clustering coefficient for each node. As comparisons, we generated a random network with a similar degree distribution in expectation and a WS small-world network (Section 2), which is known to have high clustering coefficients and short path lengths. The TEC network displayed significantly higher clustering tendency than the random graph, consistent with non-random biological organization, though lower than that of the WS model (Fig. 1f). Additionally, the TEC network showed greater variability in its node-level clustering coefficients than either model, suggesting a modular structure composed of both tightly clustered communities and sparsely connected nodes.

We also investigated the presence of rich-club organization, a phenomenon in which highly connected nodes preferentially connect to one another (Zhou and Mondragon 2004, Colizza *et al.* 2006, Zohdy *et al.* 2025). By comparing degree-specific rich-club coefficients against those of a random network with a similar distribution (Fig. 1g), we observed significantly elevated coefficients among high-degree nodes in the TEC network, indicating the presence of a rich-club organization. This observation is in contrast to protein interaction networks that tend to lack rich-club organization (Colizza *et al.* 2006, Wuchty 2007).

Finally, we computed the shortest paths between all pairs of nodes in the largest connected component of the TEC network and compared the resulting distribution to that of a WS model with the same number of nodes (Fig. 1h). The TEC network exhibited shorter average path lengths, suggesting high overall connectivity. Furthermore, combined with its moderate clustering coefficient, this suggests that the TEC network presents characteristics of a small-world topology, a common feature of biological systems (Watts and Strogatz 1998).

### 3.2 TEC network reveals distinct and complementary topological patterns and information compared to RNA co-expression network

**Figure 2.**
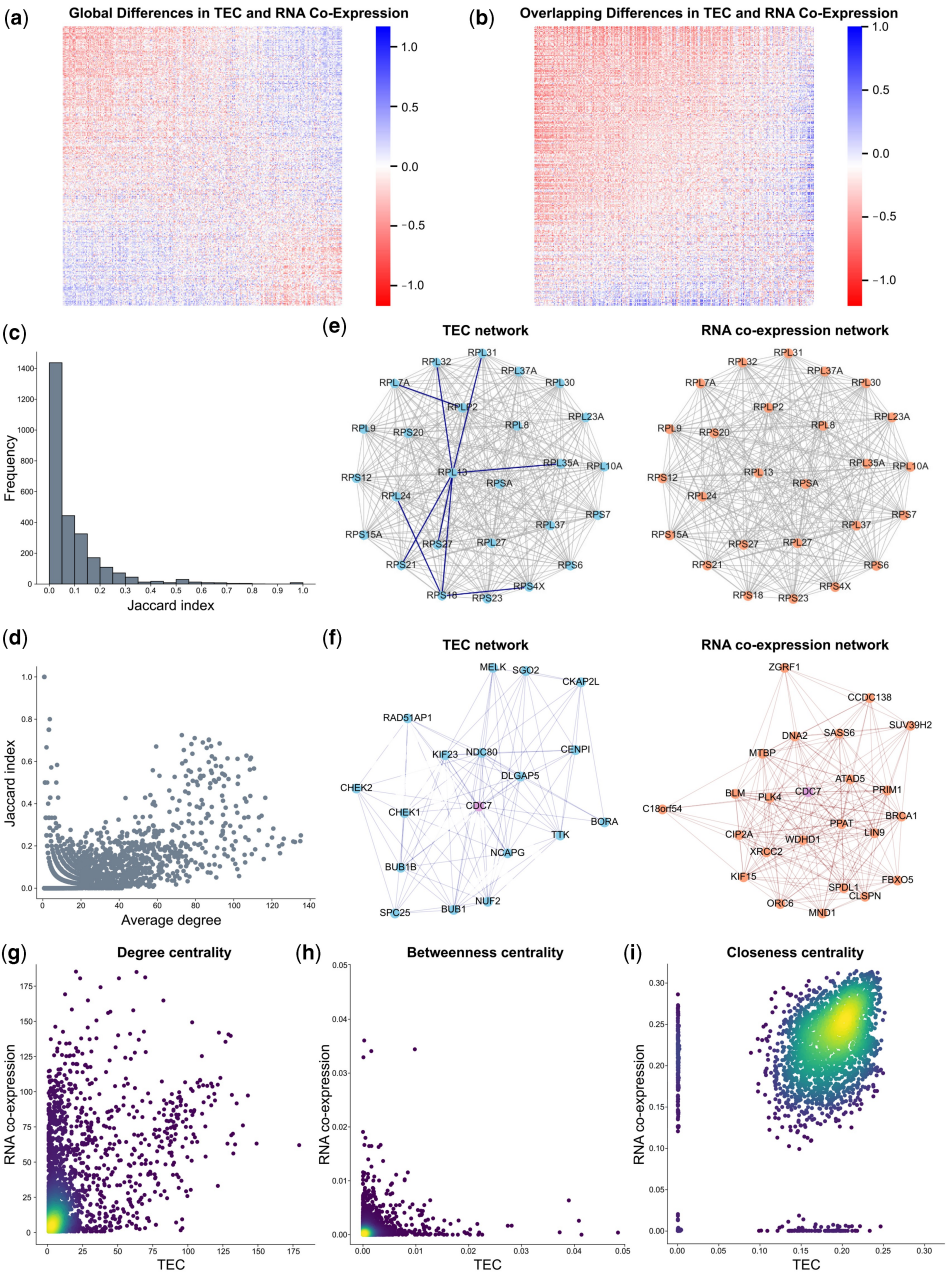
TEC network reveals distinct and complementary topological patterns and information compared to RNA co-expression network. (a) Heatmap of pairwise differences in TEC and RNA co-expression values across all 11 088 genes. (b) Heatmap of pairwise differences for overlapping genes between TEC and RNA co-expression networks. (c) Distribution of Jaccard index scores for overlapping genes between the TEC and RNA co-expression network. (d) Relationship between Jaccard index and average node degree across networks. (e) Visualization of induced subgraphs formed by ribosomal protein genes (gene names beginning with “RPS” or “RPL”) selected from among the top 45 genes ranked by Jaccard index. Shared edges between TEC and RNA co-expression networks are shown in light gray, while network-specific edges are colored dark blue (TEC) or dark orange (RNA co-expression). The strong overlap in local structure reflects topological conservation among ribosomal components. (f) Visualization of local neighborhoods centered on CDC7 (colored purple), a gene with a low Jaccard index but moderate node degree and high interconnectivity. CDC7 shows divergent topology across TEC and RNA co-expression networks. (g–i) Comparisons of network centrality measures: (g) node strength, (h) betweenness, and (i) closeness, between TEC and RNA co-expression networks for overlapping genes. Higher point density is indicated by brighter colors as estimated by the Gaussian kernel density.

We next investigated the similarities and differences between the TEC and RNA co-expression relationships derived from paired experiments (Liu *et al.* 2025). We first computed the differences between TEC and RNA co-expression values between all gene pairs and found that a substantial subset of pairs showed large discrepancies, suggesting the presence of distinct regulatory signals at the transcriptional and translational levels (Fig. 2a). This observation motivated a network-based comparison to systematically identify genes with contrasting regulatory profiles.

Similar to the TEC network, the RNA co-expression network reached its maximum number of multi-node connected components at an edge weight threshold of 0.75 (Fig. 1a, available as supplementary data at *Bioinformatics* online), which we applied to prune edges and remove isolated nodes. Unlike earlier analyses, we retained the absolute edge weights for both networks rather than binarizing them. Unless otherwise specified, all subsequent analyses were performed on these weighted graphs. Throughout, we refer to the unweighted degree of a node as its *node degree* and the sum of its edge weights as its *node strength*.

The resulting RNA co-expression network contained 4375 nodes and 52 982 edges, which forms a larger and denser network than the TEC network. This suggests that these genes are more likely to be co-expressed at the transcriptional level. We identified 2722 overlapping genes between the final TEC and RNA co-expression networks, many of which have high differences in TEC and RNA co-expression values (Fig. 2b).

To assess the similarity of local connectivity for these shared genes, we computed the Jaccard index between their respective sets of neighbors across the two networks. We found that most genes were largely connected to non-overlapping partners (Fig. 2c). Moreover, many nodes with low Jaccard index were well-connected in at least one of the two networks (Fig. 2d), reinforcing the notion that coordination at transcription and translation is largely governed by distinct mechanisms.

All genes with a Jaccard index of one, indicating identical neighborhoods in both networks, had only a single connection. Most of these gene pairs corresponded to subunits of the same macromolecular complex. Beyond these cases, we found that many of the genes with the most similar neighborhoods, particularly those with high connectivity (node degree ≥50) corresponded to components of the cytoplasmic ribosome. We visualized the induced subgraph comprising all large and small subunit ribosomal proteins among the top 45 most similar genes (Fig. 2e). The resulting subgraphs from both networks contained 23 genes each and showed substantial overlap, sharing over 300 edges with the TEC subgraph containing only nine additional edges. This strong structural similarity reflects the tightly coordinated expression of ribosomal subunits and likely represents the most structurally and functionally coherent subgraph shared between the two networks.

Given the widespread divergence in local neighborhoods across networks, we additionally computed each gene’s node degree and weighted clustering coefficient to provide insight into its centrality and the functional coherence of its local neighborhood (Langfelder and Horvath 2008, Oldham *et al.* 2008, Lewis *et al.* 2010). A full summary of these statistics is presented in Table 2, available as supplementary data at *Bioinformatics* online. One illustrative case was CDC7, which had moderate connectivity (node degree ≥10) but entirely distinct sets of neighbors: 17 in the TEC network and 23 in RNA co-expression network (Fig. 2f). Interestingly, although CDC7’s neighbors in both networks were enriched for genes involved in cell cycle regulation, neighbors in the TEC network were associated with G2/M transition and mitotic fidelity, whereas those in the RNA co-expression network were more related to DNA replication during the S phase. To ensure our results were not artifacts of the edge weight threshold, we compared each of CDC7’s retained edge weights in one network to its corresponding (excluded) weight in the other network (Fig. 2, available as supplementary data at *Bioinformatics* online). A substantial proportion of edges showed differences: 75% differed by more than 0.1 and 30% by more than 0.2, confirming genuine differences in regulatory patterns.

We next computed several centrality metrics on the set of overlapping genes to systematically analyze their topological positions across the two networks. Comparative analysis of all three centrality metrics revealed distinct patterns (Fig. 2g–i). We observed that PKM displayed a heavy imbalance in node strength (degree centrality) toward the TEC network (94.92 versus 1.55). This disparity suggests that PKM may participate in post-transcriptional regulatory processes that possibly influence ribosome coordination or modulate translation. This interpretation is supported by studies demonstrating PKM’s involvement in translational stalling (Kejiou *et al.* 2023).

Conversely, PSMA5, a subunit of the 26S proteasome, showed the greatest degree of centrality imbalance in favor of the RNA co-expression network (185.32 versus 20.25). Similar patterns of imbalance were observed in other 26S proteasomal subunits, such as PSMA4, PSMB5, and PSMB6. This is consistent with known co-regulation of proteasomal components at the transcriptional level by shared transcription factors (Motosugi and Murata 2019, Gilda *et al.* 2024). Overall, our results demonstrate that the RNA co-expression and TEC networks capture many distinct patterns, revealing complementary insights into the organization of regulatory interactions.

### 3.3 TEC network is enriched with biological signals and organizes genes into functionally coherent clusters

**Figure 3.**
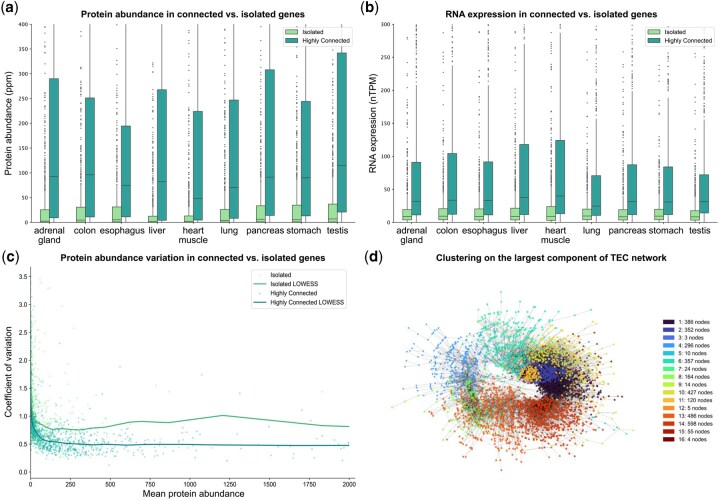
TEC network is enriched with biological signals and organizes genes into functionally coherent clusters. (a and b) Box-and-whisker plot comparing protein abundance (a) and RNA expression (b) between the top 1000 most connected genes (dark green) and 1000 randomly selected, isolated genes (light green). Boxes represent the interquartile range (IQR), with whiskers extending to [Q1−1.5×IQR,Q3+1.5×IQR]. (c) Comparison of tissue protein abundance coefficient of variation as a function of mean abundance between the top 1000 highly connected genes (dark green) and 1000 randomly selected isolated genes (light green). Curves were fitted using locally weighted scatterplot smoothing (LOWESS) to highlight overall trends. (d) Clustering of the largest connected component of the TEC network using the Louvain algorithm. The network was visualized using the Kamada–Kawai force-directed layout (Kamada and Kawai 1989), and the number of nodes in each cluster is listed.

We next evaluated whether genes with higher connectivity in the TEC network exhibit elevated and more consistent expression across cell types. To test this, we compiled RNA expression (Uhlén *et al.* 2015) and protein abundance data (Huang *et al.* 2023) for the top 1000 most connected genes and compared them to 1000 randomly selected, isolated genes across nine tissues. As anticipated, highly connected genes exhibited significantly greater protein abundance and RNA expression levels across all nine tissues, with at least a 10-fold higher median protein abundance and a two-fold increase in median RNA expression (Fig. 3a and b). This pattern is not observed when comparing two randomly sampled gene sets.

While these findings indicate that highly connected genes tend to be more abundantly expressed, they do not necessarily imply reduced tissue specificity. To assess this, we analyzed expression variability by computing the coefficient of variation (CV) across tissues for both gene sets. To control for differences in expression level, we organized genes by their mean expression level. Highly connected genes displayed lower variability in protein abundance data, indicating a broader, less tissue-specific expression pattern (Fig. 3c). Specifically, among genes with mean abundance above 50 parts per million, the median CVs, computed across 10 equal-sized bins by mean abundance, were on average 1.5-fold higher in isolated genes compared to connected genes. This trend is consistently observed for RNA expression data (isolated genes showing an average of 1.4-fold higher median CVs across bins for mean expression above 15 normalized transcripts per million; Fig. 3a, available as supplementary data at *Bioinformatics* online) but was not observed in randomly sampled gene sets.

To explore the biological relevance of highly connected central nodes, we examined the top ten genes ranked by node strength (Fig. 3c and d, available as supplementary data at *Bioinformatics* online). Three of these genes, CCT3, CCT4, and CCT8, are subunits of the chaperonin containing TCP-1 (CCT) complex. The CCT complex maintains protein homeostasis by assisting the folding of nascent polypeptide chains in eukaryotic cells, a process essential for proper protein synthesis (Que *et al.* 2024). Additionally, CCT3 and CCT4 also exhibited a strong node degree imbalance favoring the TEC network and were highly expressed across tissues at both RNA and protein levels.

In GCNs, densely connected gene groups often form modules that participate in shared biological activities (Stuart *et al.* 2003, Mao *et al.* 2009, Kuang *et al.* 2022). Among these, cliques represent the most cohesive network units (Voy *et al.* 2006). We identified all maximal cliques in the TEC network using the Bron–Kerbosch algorithm (Bron and Kerbosch 1973) (Fig. 3b and Table 3, available as supplementary data at *Bioinformatics* online). The largest clique contained 75 genes and consisted largely of ribosomal protein genes. As expected, this clique was strongly enriched for translation-related functions, as confirmed by Gene Ontology (GO) enrichment analysis (Ashburner *et al.* 2000, Aleksander *et al.* 2023) on the Biological Process aspect via EnrichR (Xie *et al.* 2021) (28 terms with Benjamini-Hochberg-adjusted *P*-values <.05 and odds ratios >10; Table 4, available as supplementary data at *Bioinformatics* online).

We next applied unsupervised clustering to the largest connected component of the TEC network using the Louvain algorithm (Blondel *et al.* 2008). The algorithm produced 16 clusters, with sizes ranging from 4 to 642 nodes (Fig. 3d, Table 5, available as supplementary data at *Bioinformatics* online). One particular cluster included genes ALG8, DERL2, DRAM2, C5orf15, TMEM41B, YIPF5, GOLT1B, MSMO1, SC5D, and SEC23A. With the exception of DRAM2, the encoded proteins are all localized to the endoplasmic reticulum (ER) and/or Golgi apparatus. Consequently, the GO term “ER membrane” (GO:0005789) was the most significantly enriched (adjusted *P*-value = .00013; an odds ratio of 22.61). While recent work (Horste *et al.* 2023) showed that ER-localized transcripts display coordinated and differential translation patterns in a single-cell line, our results suggest that such subcytoplasmic compartmentalization of translation may represent a more generalizable regulatory mechanism across cell types.

### 3.4 Tissue-specific TEC networks reveal contextual co-translational patterns

Our analyses thus far have focused on a global TEC network constructed using samples derived from a wide range of cell types. Consequently, it does not reflect tissue-specific variations, which can uncover additional insights into tissue-specific functional relationships (Guan *et al.* 2012, Greene *et al.* 2015). To explore this possibility, we calculated liver, lung, and brain-specific TEC by grouping cell lines from Ribobase according to their tissue of origin (Section 2). These tissues were selected due to their relatively high representation in the dataset.

**Figure 4.**
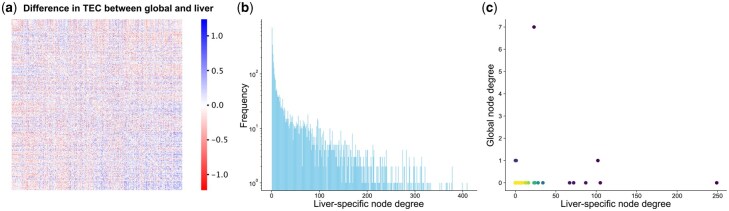
Tissue-specific TEC networks reveal contextual co-translational patterns. (a) Differences between global TEC values and liver-specific TEC values for overlapping genes across the two dataset. (b) Degree distribution of the liver-specific TEC network after removing isolated nodes using a cutoff of 0.75. The *y*-axis is log-scaled. (c) Comparison of node degree for liver-specific genes between the global and liver-specific TEC networks.

Our analysis primarily focused on the liver-specific TEC dataset, which contained the largest number of samples and was expected to yield the most robust results. This dataset comprised 10 101 genes, of which 9477 overlapped with those in the global dataset. To assess divergence, we calculated the pairwise differences in TEC values between the global and liver-specific datasets (Fig. 4a). We observed considerable differences among the overlapping genes: 29% of differences exceeded 0.25 and 3% exceeded 0.5. Similar trends were observed across the other two datasets, with the brain-specific network displaying the greatest divergence (48% above 0.25 and 16% above 0.5; Fig. 5a, available as supplementary data at *Bioinformatics* online). These findings suggest that networks constructed from tissue-specific data may capture regulatory dynamics not reflected in the global model.

To construct the liver-specific TEC network, we applied the same edge weight threshold of 0.75 used in the global network to ensure comparability. The resulting network contained 3, 784 nodes and 78 964 edges (Fig. 4b). In contrast to the global network, more than 30 genes in the liver-specific network had over 300 connections. This phenomenon is even more pronounced in the lung and brain-specific networks, where multiple nodes have over 600 connections (Fig. 5b, available as supplementary data at *Bioinformatics* online). Additionally, all three tissue-specific networks were at least two times denser than the global network (Section 2). This increase in density persists across different thresholds and likely reflects the ability of tissue-specific TEC to capture specific relationships that are diluted in the global context. Among the three, the brain-specific network is approximately three times as dense as the liver-specific network at the 0.75 threshold, suggesting more brain-specific co-translational relationships are present within our dataset.

We next examined whether genes specifically expressed in a given cell type also form strong TEC partnerships restricted to that same cell type. To identify such cell-type-specific genes, we referenced Human Protein Atlas (Uhlén *et al.* 2015), which defines *tissue-enriched* genes as those with at least a fourfold higher expression in the given tissue relative to all other tissues, and *tissue-enhanced* genes as those with at least a fourfold higher tissue expression compared to the mean expression across all tissues. Based on these, we classified genes as *liver-specific* if they demonstrated either liver tissue-enriched or tissue-enhanced behavior in both RNA expression and protein abundance data. A total of 57 liver-specific genes were shared between the global and liver-specific TEC networks. With the exception of one gene, all of these genes had lower connectivity in the global network as compared to the liver-specific network (Fig. 4c).

Among these, PHYH demonstrated the most pronounced difference in connectivity with 249 connections in the liver-specific network and none in the global network. PHYH encodes a peroxisomal protein involved in the alpha-oxidation of 3-methyl branched fatty acid, a key step in the catabolism of phytanic acid (Brown *et al.* 2015). The elevated connectivity of PHYH in the liver-specific network, which is absent in the global network, is consistent with the function of the liver in fatty acid metabolism.

## 4 Discussion

In this study, we systematically characterized a biological network constructed using TEC as introduced by Liu *et al.* (2025). The selection of an appropriate edge weight threshold can substantially influence both network topology and downstream analyses. Numerous thresholding strategies have been proposed, including statistical significance-based frameworks (Reverter and Chan 2008), soft thresholding (Zhang and Horvath 2005), and methods guided by network metrics (Zhang and Horvath 2005, Elo *et al.* 2007). Each approach involves trade-offs between statistical rigor, biological relevance, and computability (Borate *et al.* 2009, Perkins and Langston 2009). Here, we employed a simple connected component-based thresholding technique that retained edges indicative of high confidence relationships that likely reflect coordinated regulation. To reduce potential artifacts introduced by the threshold selection, we performed supplementary analyses to assess the robustness of our results where relevant (Tables 1 and 6 and Fig. 4, available as supplementary data at *Bioinformatics* online).

We compared the TEC network to a conventional RNA co-expression network constructed from the same set of genes. Our analysis revealed that two networks differ substantially in their topological structures with each capturing both shared and distinct aspects of cellular regulatory architectures. Although not explored in this study, we speculate a similar case may hold for protein co-regulation networks constructed from protein co-expression analysis (Kustatscher *et al.* 2019). These findings motivate the future development of data aggregation methods, which may yield better results in downstream tasks such as function or interaction prediction.

We observed that gene connectivity within the TEC network correlates with RNA and protein abundance, as well as tissue specificity. Consequently, relationships involving genes with low expression or tissue-specific enrichment are likely to be missed in the global network analysis. We attempted to address this limitation by constructing and analyzing tissue-specific TEC networks. We found that these networks tend to be more densely connected than their global counterpart, likely reflecting study-specific or tissue-specific relationships that became diluted in the overarching global network. Furthermore, as an example, we demonstrated that the liver-specific network could uncover gene functions not detected in the global network, highlighting the utility of tissue-specific analyses.

A key limitation of our study is the relatively small size of our dataset. This constraint is further intensified in the construction of tissue-specific TEC networks, which rely on only around 25–90 samples for TEC estimation. This limit in dataset size, combined with our focus on a single species, may hinder the generalizability of our findings. Furthermore, obtaining TEC values requires either experimental sequencing or manual curation from publicly available sequencing databases, both of which are time-consuming and resource-intensive. This significantly constrains our ability to scale up the network. A potential solution to this challenge is the development of computational models capable of accurately predicting mRNA expression levels (Linder *et al.* 2025) and TE values (Zheng *et al.* 2025), which could enable broader application of our framework without the need for extensive experimental data. As single-cell ribosome profiling (Ozadam *et al.* 2023, Rao *et al.* 2025) becomes more widely adopted, graph-based TEC analyses could similarly reveal heterogeneity in translational regulation at single-cell resolution.

## Supplementary Material

btaf583_Supplementary_Data

## Data Availability

The code for this study is archived on https://zenodo.org/records/17275939 and publicly available at https://github.com/CenikLab/TEC-Network-Analyses. The associated data can be accessed at https://zenodo.org/records/17275970.
