# Supplementary material for: Topological and functional characterization of human translation efficiency covariation network
Source: Bioinformatics. 2025 Oct 22;41(11):btaf583. doi: 10.1093/bioinformatics/btaf583 (PMC12596699; doi:10.1093/bioinformatics/btaf583)
Supplement: btaf583_Supplementary_Data [file btaf583_supplementary_data.zip › TEC Network Supplementary.pdf]

SUPPLEMENTARY DATA

Supplementary Figures

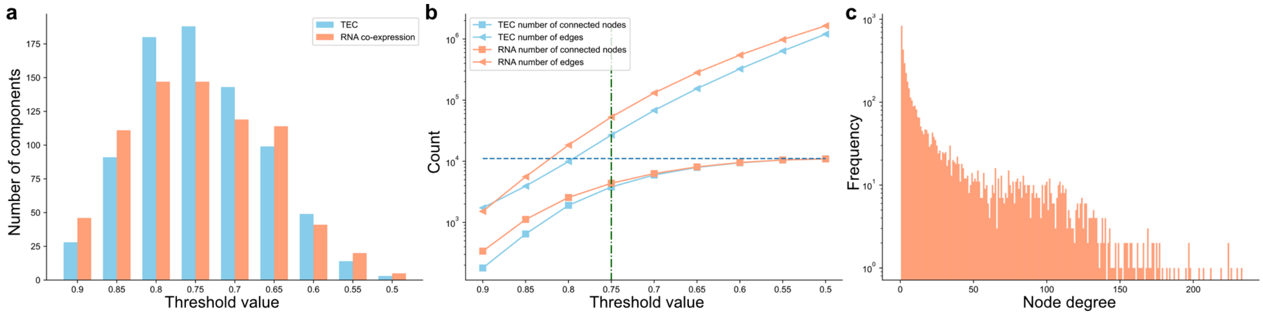

Supplementary Figure 1. Edge weight threshold comparison and RNA co-expression network distribution. a, Number of multi-node connected components in the TEC (blue) and RNA co-expression (orange) networks across varying edge weight thresholds. **b,** Number of nodes (squares) and edges (triangles) retained in the TEC (blue) and RNA co-expression (orange) networks across varying edge weight thresholds. **c,** Unweighted node degree distribution of the RNA co-expression network after removing isolated nodes using a cutoff of 0.75. The y-axis is log-scaled to account for variability in node connectivity.

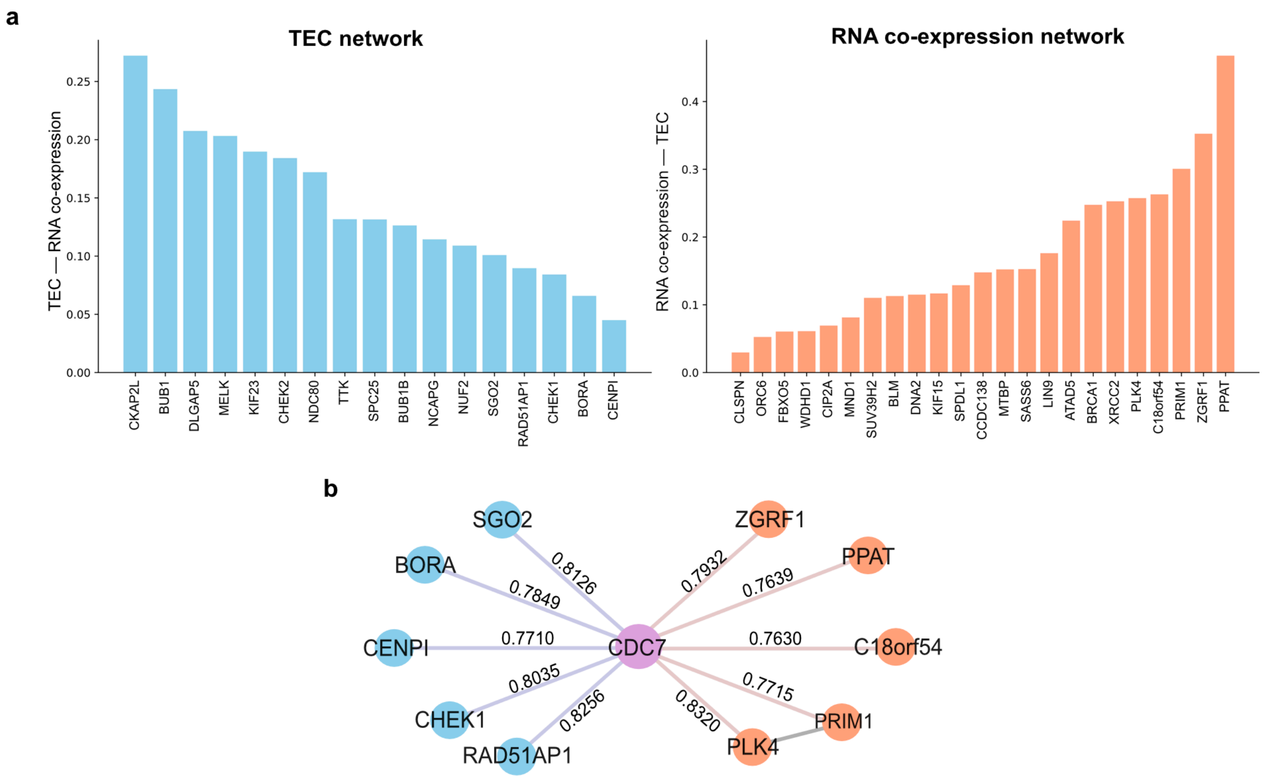

Supplementary Figure 2. *CDC7* exhibits distinct connectivity patterns between the TEC and RNA co-expression networks. **a,** Difference in edge weights for CDC7's connections across the two networks. **b,** Top five strong connections for CDC7 in each network. Gray edges indicate connections with weights greater than 0.85.

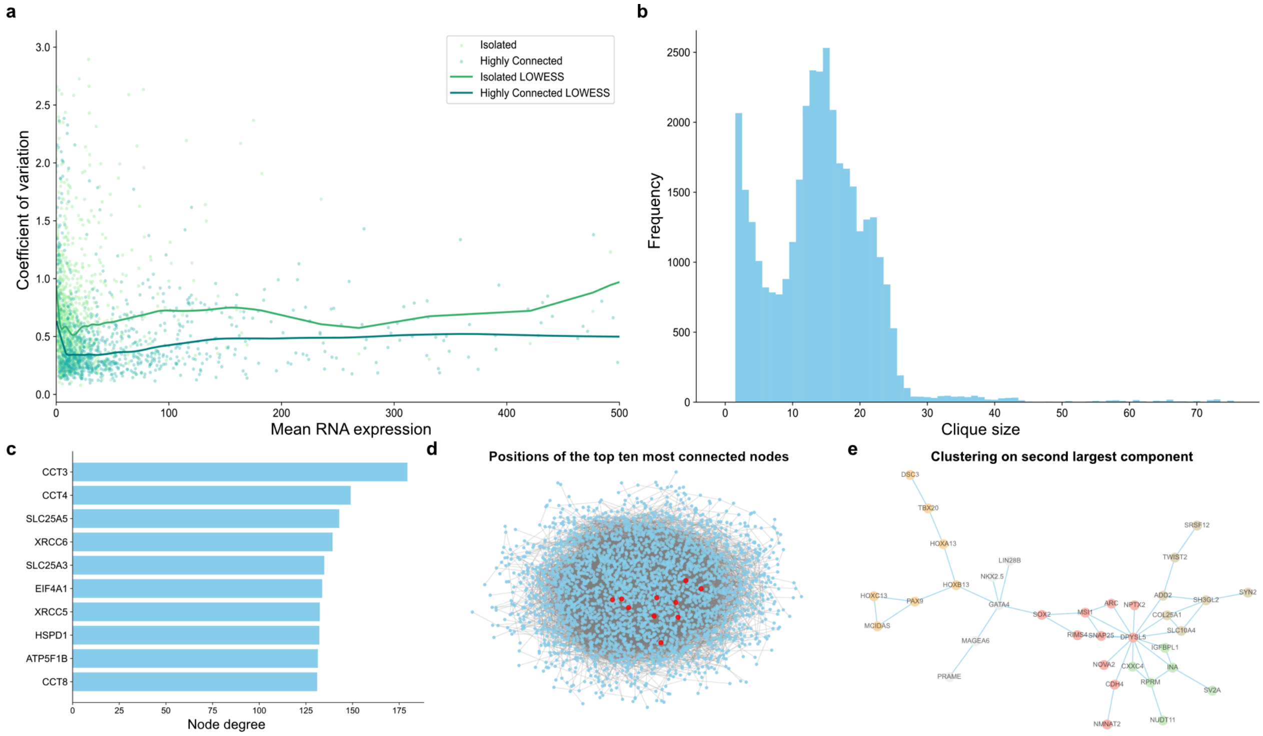

Supplementary Figure 3. a, Comparison of coefficient of variation of tissue RNA expression as a function of mean expression between the top 1,000 highly connected genes (dark green) and 1,000 randomly selected isolated genes (light green). Smoothed curves were fitted using LOWESS. **b,** Distribution of maximal cliques sizes in the TEC network. **c,** Top ten nodes in the TEC network ranked by node strength. **d,** Network positions of the top ten most connected nodes in the TEC network. **e,** Louvain clustering of the second largest connected component of the TEC network. The network was visualized using the Kamada–Kawai force-directed layout, with nodes labeled by their corresponding gene symbols.

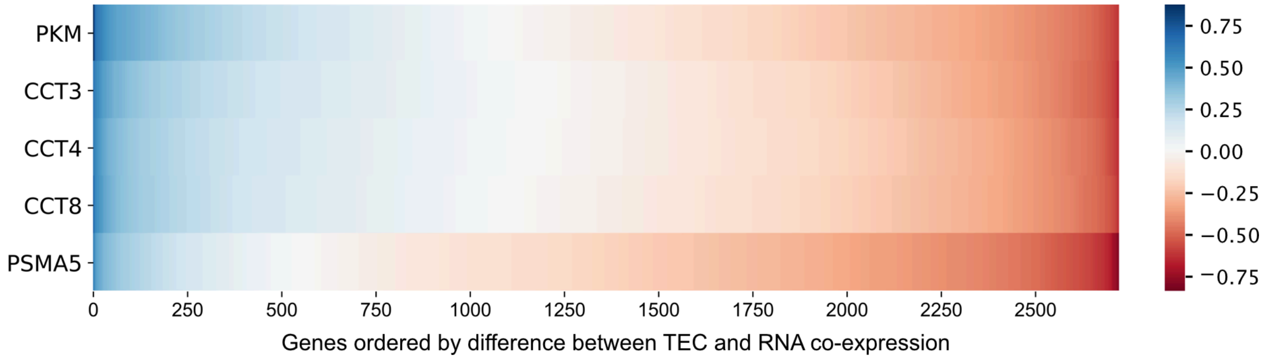

Supplementary Figure 4. Pairwise differences between TEC and RNA co-expression values for all genes explicitly discussed as showing degree node or strength imbalance toward either the TEC or RNA co-expression network. Each row is independently sorted in descending order.

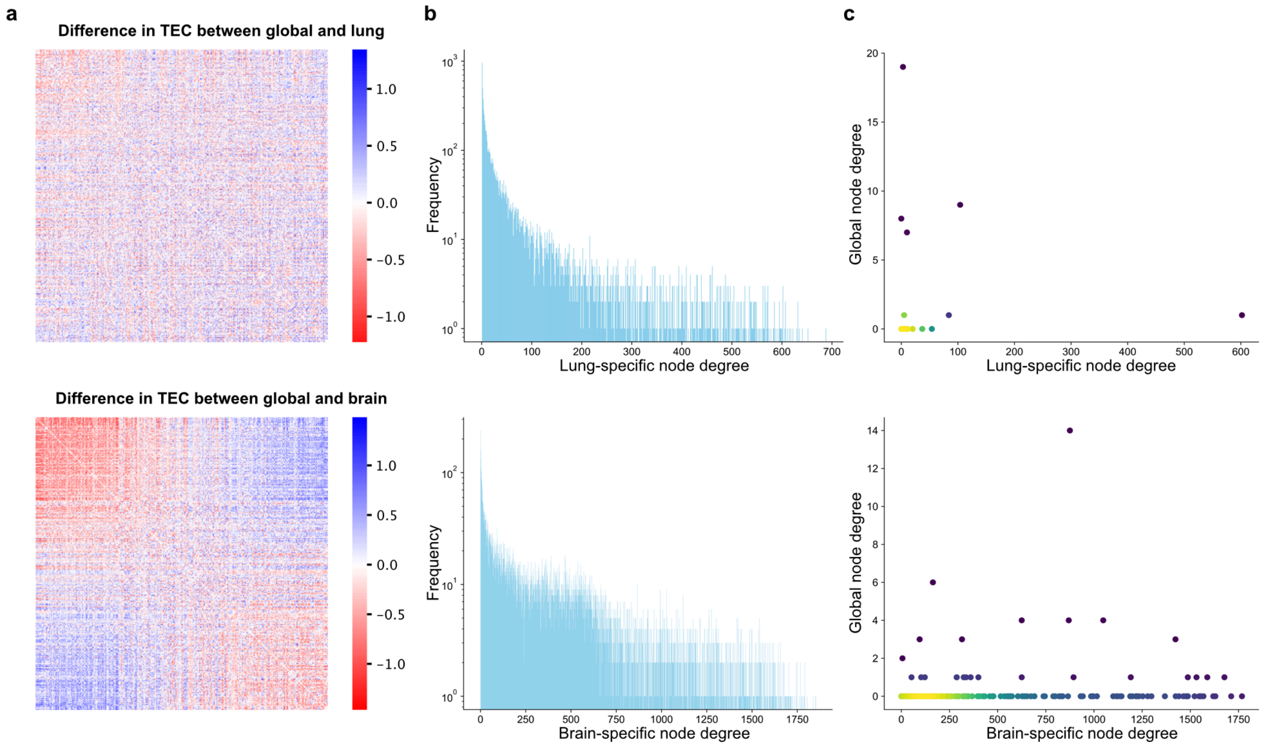

Supplementary Figure 5. a, Differences between global TEC values and tissue-specific TEC values for genes present in both datasets. The top panel shows lung-specific data and bottom panel shows brain-specific data. **b,** Degree distribution of the lung-specific (top) and brain-specific TEC (bottom) networks after removing isolated nodes using a cutoff of 0.75. The y-axis is log-scaled. **c,** Comparison of node degree for lung-specific genes between the global and lung-specific TEC networks (top), and for brain-specific genes between the global and brain-specific TEC networks (bottom).

Supplementary Tables

- **Table S1.** Scale-free analysis of the TEC network across edge weight thresholds.
- **Table S2.** Jaccard index, node degree, and clustering coefficient for genes shared between the TEC and RNA co-expression networks. Rows are sorted by the mean clustering coefficient across both networks.
- **Table S3.** List of cliques in the TEC network, ordered by size.
- **Table S4.** GO Biological Process enrichment analysis (via EnrichR) of genes in the largest maximal clique of the TEC network.
- **Table S5.** Gene names and their corresponding clusters, as shown in Figure 3d, within the TEC network at a threshold of 0.75.
- **Table S6.** Global clustering coefficients and average shortest path lengths of the TEC network across edge weight thresholds.
